# Altered dopaminergic firing pattern and novelty response underlie ADHD-like behavior of SorCS2-deficient mice

**DOI:** 10.1038/s41398-021-01199-9

**Published:** 2021-01-25

**Authors:** Ditte Olsen, Niels Wellner, Mathias Kaas, Inge E. M. de Jong, Florence Sotty, Michael Didriksen, Simon Glerup, Anders Nykjaer

**Affiliations:** 1 Department of Biomedicine, Aarhus University, Hoegh-Guldbergsgade 10, DK-8000 Aarhus C, Denmark; 2 Neurodegeneration and Biologics, H. Lundbeck A/S, Ottiliavej 9, DK-2500 Valby, Denmark; 3 Danish Research Institute of Translational Neuroscience DANDRITE Nordic-EMBL Partnership, Department of Biomedicine, Aarhus University, Hoegh-Guldbergsgade 10, DK-8000 Aarhus C, Denmark; 4 The Danish National Research Foundation Center PROMEMO, Aarhus University, Hoegh-Guldbergsgade 10, DK-8000 Aarhus C, Denmark; 5 Department of Neurosurgery, Skejby University Hospital, Palle Juul-Jensens Blvd. 99, DK-8200 Aarhus N, Denmark; 6 Present Address: Department of Biomedicine, Aarhus University, Hoegh-Guldbergsgade 10, DK-8000 Aarhus C, Denmark

**Keywords:** ADHD, Molecular neuroscience

## Abstract

Attention deficit hyperactivity disorder (ADHD) is the most frequently diagnosed neurodevelopmental disorder worldwide. Affected individuals present with hyperactivity, inattention, and cognitive deficits and display a characteristic paradoxical response to drugs affecting the dopaminergic system. However, the underlying pathophysiology of ADHD and how this relates to dopaminergic transmission remains to be fully understood. *Sorcs2*^*−/−*^ mice uniquely recapitulate symptoms reminiscent of ADHD in humans. Here, we show that lack of SorCS2 in mice results in lower sucrose intake, indicating general reward deficits. Using in-vivo recordings, we further find that dopaminergic transmission in the ventral tegmental area (VTA) is shifted towards a more regular firing pattern with marked reductions in the relative occurrence of irregular firing in *Sorcs2*^*−/−*^ mice. This was paralleled by abnormal acute behavioral responses to dopamine receptor agonists, suggesting fundamental differences in dopaminergic circuits and indicating a perturbation in the balance between the activities of the postsynaptic dopamine receptor DRD1 and the presynaptic inhibitory autoreceptor DRD2. Interestingly, the hyperactivity and drug response of *Sorcs2*^*−/−*^ mice were markedly affected by novelty. Taken together, our findings show how loss of a candidate ADHD-risk gene has marked effects on dopaminergic circuit function and the behavioral response to the environment.

## Introduction

Attention deficit hyperactivity disorder (ADHD) is characterized by symptoms of inattention, hyperactivity, and impulsivity^1,2^. It is the most frequently diagnosed neurodevelopmental disorder affecting 5% of children and adolescents as well as 2.5% of adults worldwide but the etiology and pathophysiology are poorly understood. Brain imaging, post mortem studies, genetics, and the abnormal behavioral response of patients to drugs affecting the dopaminergic system clearly implicate altered dopaminergic transmission as a key factor in this disorder. In fact, amphetamine and analogs, which cause an increase in synaptic dopamine, are currently the most effective treatment of ADHD^2^.

ADHD is among the most highly inheritable neurological disorders with a heritability factor of 70–80%^2–4^. Several genes have been proposed to play a role in the development^5^ but no single major ADHD-risk gene has been found so far, suggesting that ADHD for the most part is a polygenic disorder. However, multiple rare and functionally disruptive variants, may cause ADHD too. Elucidating the mode of action of these genes and what goes wrong when disrupted are fundamental to understand the molecular mechanisms underlying ADHD. *SORCS2* has been associated with risk of ADHD^6,7^ in addition to bipolar disorder^8–11^, schizophrenia^10^, and symptoms of alcohol withdrawal^12^. We and others have shown that SorCS2 influences neurite outgrowth of CNS neurons^13–15^. In particular, SorCS2 is expressed during embryogenesis in dopaminergic precursors of the ventral mesencephalon that develop into the ventral tegmental area (VTA) and substantia nigra. Midbrain explants from *Sorcs2*^*−/−*^ mouse embryos exhibit increased outgrowth of tyrosine-hydroxylase expressing (TH+) projections and in adult *Sorcs2*^*−/−*^ mice the frontal cortex is hyperinnervated, arguing for a critical role of SorCS2 in growth cone retraction during dopaminergic innervation. As a consequence, *Sorcs2*^*−/−*^ mice are hyperactive, risk-taking, and inattentive; key traits of ADHD^11,15^. Further, treatment with amphetamine at a dose that induces markedly increased locomotor activity in wild-type (Wt) animals normalized motor activity in the *Sorcs2*^*−/−*^ mice^15^.

In the present study, we show that lack of SorCS2 results in fundamental changes in the functionality of the dopaminergic system. Furthermore, the hyperactivity and response to dopamine agonists in *Sorcs2*^*−/−*^ mice is remarkably sensitive to novelty. Our findings provide insights into how a psychiatric risk gene affects dopaminergic circuit function and the response to central stimulants.

## Materials and methods

### Animals

The generation of *Sorcs2*^*−/−*^ mice has been described previously^15^. All experiments were performed on adult (older than 12 weeks old) C57Bl/6J BomTac and *Sorcs2*^*−/−*^ male mice (except for sucrose preference test were 5 female mice of each genotype were also tested with similar results as the males. Only results for the males are shown). The *Sorcs2*^*−/−*^ mice had been backcrossed on a C57Bl/6J BomTac background for 10 generations, before used for testing. The resulting *Sorcs2*^*−/−*^ mice displayed similar behavioral phenotype as those obtained through heterozygous breeding of *Sorcs2*^*+/−*^ mice. The mice were housed in groups, and kept in conditions of constant temperature (22 ± 1.5 °C), humidity (55–65%), and light/dark cycle (light on from 6.00 am to 6.00 pm) with food and water available ad libitum. The experiments were conducted between 7 am and 4 pm. Group size for each experiment was 6–12 mice of each genotype. The group size was determined based on previously published differences in locomotor activity between Wt and *Sorcs2*^*−/−*^ mice^15^. Wt and *Sorcs2*^*−/−*^ mice were consistently tested in parallel. Naïve animals were used for each experiment and no mice were used in sequential tests. All experiments were approved by the Danish Animal Experiment Inspectorate under the Ministry of Justice and complied with Danish and European regulations concerning experimentation and care of experimental animals (2012-15-2934-00397, 2016-15-0201-01127).

### Sucrose preference test

The mice were microchipped (Datamars slim transponder, Kruuse, Denmark) by anaesthetizing the animal using isoflurane, making a small cut into the neck skin, inserting the microchip, and closing the wound with indermil xfine (Henkel, Germany).

The mice (*n* = 4 of each genotype) were kept group-housed in an automated water-intake monitoring system (HM-2 system, MBRose, Denmark) in the same groups as their home-cage (4 animals per cage). The mice were habituated to the HM-2 system cages and to the presence of two drinking bottles for two days, where one of the drinking bottles was exchanged for a bottle containing a 4% sucrose solution. The position of the two drinking bottles were switched every 24 h at 11.00 am. The water and sucrose intake were monitored for three days.

### In vivo single-unit recording of dopaminergic neurons in VTA

In vivo single-unit recording of dopaminergic neurons was performed as previously described^16^. Mice were anesthetized by an initial intraperitoneal injection of urethane (1.2 g/kg), which provided long-lasting, irreversible anesthesia. If necessary, the mice received an additional dose of urethane during the recording session. The body temperature of the mouse was maintained at 37.5 °C during the entire experiment using a heating pad. A mouse was mounted in a stereotaxic frame, the skull was exposed, and a hole was drilled above the VTA. Borosilicate glass capillary was pulled to a fine tip and filled with 2% Pontamine Sky Blue in 0.5 M sodium acetate and broken back to achieve a final in vitro impedance at 3–5 MΩ at 135 Hz. The electrode was then lowered into the dorsal border of the VTA using a motorized micromanipulator (single axis IVM, Scientifica, Uckfield, UK). Recordings were obtained at the following coordinates, according to the atlas of Franklin and Paxinos:^17^ −2.8 to −3.6 mm posterior to Bregma and 0.3–0.7 mm lateral to midline. Presumed dopaminergic neurons were characterized by a slow firing pattern (0.5–10 Hz) and a triphasic action potential with a predominant positive component, a negative component followed by a minor positive component, with an overall duration >2.5 ms^18^. Extracellular action potentials were amplified, discriminated, and monitored on an oscilloscope and an audiomonitor (AM-10, Grass Technologies, West Warwick, USA). Neurons were recorded and analyzed using Spike 2 software (Cambridge Electronic Design Ltd., Cambridge, UK). Each spontaneously active dopaminergic neuron was recorded for a minimum of three minutes for offline analysis. When the experiment was completed, the last recorded site was marked by iontophoretic ejection of 2% Pontamine Sky Blue. The mice were then decapitated and their brain removed and sectioned for histological verification of the position of the electrode.

The basal firing rate and the coefficient of variation of the interspike interval (CVisi), were determined for each recorded neuron using Spike 2 Software and a built-in script (Spike2, CED, Cambridge, UK). The CVisi, which is an indicator of spiking regularity (the lower CVisi, the more regular firing), was defined as the ratio between the average interspike interval (ISI) and the standard deviation of the ISI × 100. In addition, the neuronal firing pattern of each neuron was classified as regular, irregular, or bursty based on the construction of autocorrelograms as described previously^19^. Briefly, autocorrelograms were constructed from spike trains consisting of at least 500 consecutive spikes using a bin width of 10 ms for intervals up to 2000 ms and were used to qualitatively classify neurons as firing in the regular, irregular, or bursty firing pattern. Autocorrelograms showing 3 or more regularly occurring peaks were characteristic of the regular firing pattern. An initial trough that rose smoothly to a steady state was classified as irregular firing pattern while an initial peak followed by decay to a steady-state was classified as bursty firing pattern. To further confirm the classification of the firing pattern, another method based on the construction of discharge density histograms was used, as described elsewhere^20^. The spike density corresponds to the number of spikes in a time interval which duration is calculated based on the average firing rate. Spike density histograms showing 3–5 symmetrical organized bins were indicative of a regular firing pattern (the sum of the 1–2 smallest should be less than 1% of the largest bin). A maximum of six asymmetrically organized bins resulted in an irregular firing pattern. Finally, six or more bins with a maximum spike count for the first bin were classified as a bursty firing pattern.

48 Wt neurons were recorded (from 10 mice) and 63 neurons were recorded in *Sorcs2*^*−/−*^ mice (from 12 animals).

### Locomotor activity

The experiment was conducted as previously described^21^. Briefly, the mice were transported from the stable to the laboratory one day prior to the test. On the test day, mice were placed in individual test cages (macrolon type III, 382 mm × 220 mm × 150 mm), which were placed in a U-frame equipped with two rows of four infrared light sources and photocells. Recording of an activity count required the interruption of two adjacent light beams in the lower row thereby avoiding counts induced by stationary movements. A rearing count required the interruption of two light beams located above each other. The registration and timing of locomotor and rearing activity were fully automated by custom-designed hardware and software by Ellegaard Systems A/S, Faaborg, Denmark.

The mice were weighed and injected with saline (0.9%), amphetamine (1.25, 2.5, 5, or 10 mg/kg), cocaine (10, 20, or 30 mg/kg), SKF-38393 (3, 10 or 30 mg/kg), or quinpirole (0.0025, 0.01 or 0.04 mg/kg). The mice were either injected immediately before placing them in the test cage and monitored for one hour, or the mice were put in the test cage and monitored for an hour prior to injection and subsequently monitored for an additional hour.

The same equipment was used for testing the mice in their home-cage environment for 120 h. The mice were single-housed during this experiment, and there were no nesting material in the cages.

For testing the effect of methylphenidate, an open field test was conducted. The mice were habituated to a clear Plexiglas arena (40 cm × 40 cm × 35 cm) prior to injection with saline or methylphenidate (3, 5, or 10 mg/kg) whereafter being placed back into the Plexiglas arena where their activity was recorded over a 40 min session and analyzed using the Any-maze tracking software (Stoelting Europe) (Table 1).

**Table 1:** Number of mice used for each of the locomotor studies.

Experiment	Wt	*Sorcs2*^*−/−*^
General locomotor activity	48	48
Home-cage activity	8	8
Amphetamine (with habituation phase)	12	12
Amphetamine (without habituation phase)	12	12
Cocaine	10–12	10–12
Methylphenidate	6	6
SKF38393 (with habituation phase)	6	6
SKF38393 (without habituation phase)	6	6
Quinpirole	6	6

### Drugs

d-Amphetamine (Sigma-Aldrich), cocaine (Sigma-Aldrich), SKF-38393 (Sigma-Aldrich), methylphenidate (Sigma-Aldrich), and quinpirole (Sigma-Aldrich) were dissolved in 0.9% saline. Drugs were selected based on their documented effect on the rodent dopaminergic system and their known mechanism of action. The dose was selected based on previously published behavioral studies in mice^21–26^.

Drugs were administrated subcutaneously (sc.), in a volume of 10 ml/kg, unless otherwise stated.

### Statistics

Statistical analysis was conducted using Prism 6.0 for Mac, GraphPad Software, La Jolla California USA. Sample sizes were chosen on the basis of previous experience with similar experiments. While the researchers were not blinded in the experiments, all intake and activity (except from dopaminergic activity in the in vivo electrophysiology) were sampled automatically. No animals were excluded for statistical analysis. For each experiment, statistical analysis method, sample size, and p-values are provided in the figure legends. The used statistical tests are justified as appropriate and the data meet the assumptions of the test. Statistical comparisons were made by Student’s *t*-test (unpaired, two-sided), Mann-Whitney test, Chi-square test, one-way, two-way ANOVA or mixed ANOVA (when analysis with repeated measurement over time were made) followed by *post hoc* Tukey’s or Sidak’s multiple comparison test. Non-parametric statistics were performed for experiments in which at least one group demonstrated non-normal distribution. Each experiment was conducted once in the groups of the stated sizes (*n*). Data are presented as mean ± standard error of the mean (S.E.M) and probability (*p*) values of less than 5% were considered statistically significant.

## Results

### *Sorcs2*^*−/−*^ mice show altered in-vivo firing pattern of dopaminergic neurons in the VTA

**Fig. 1:**
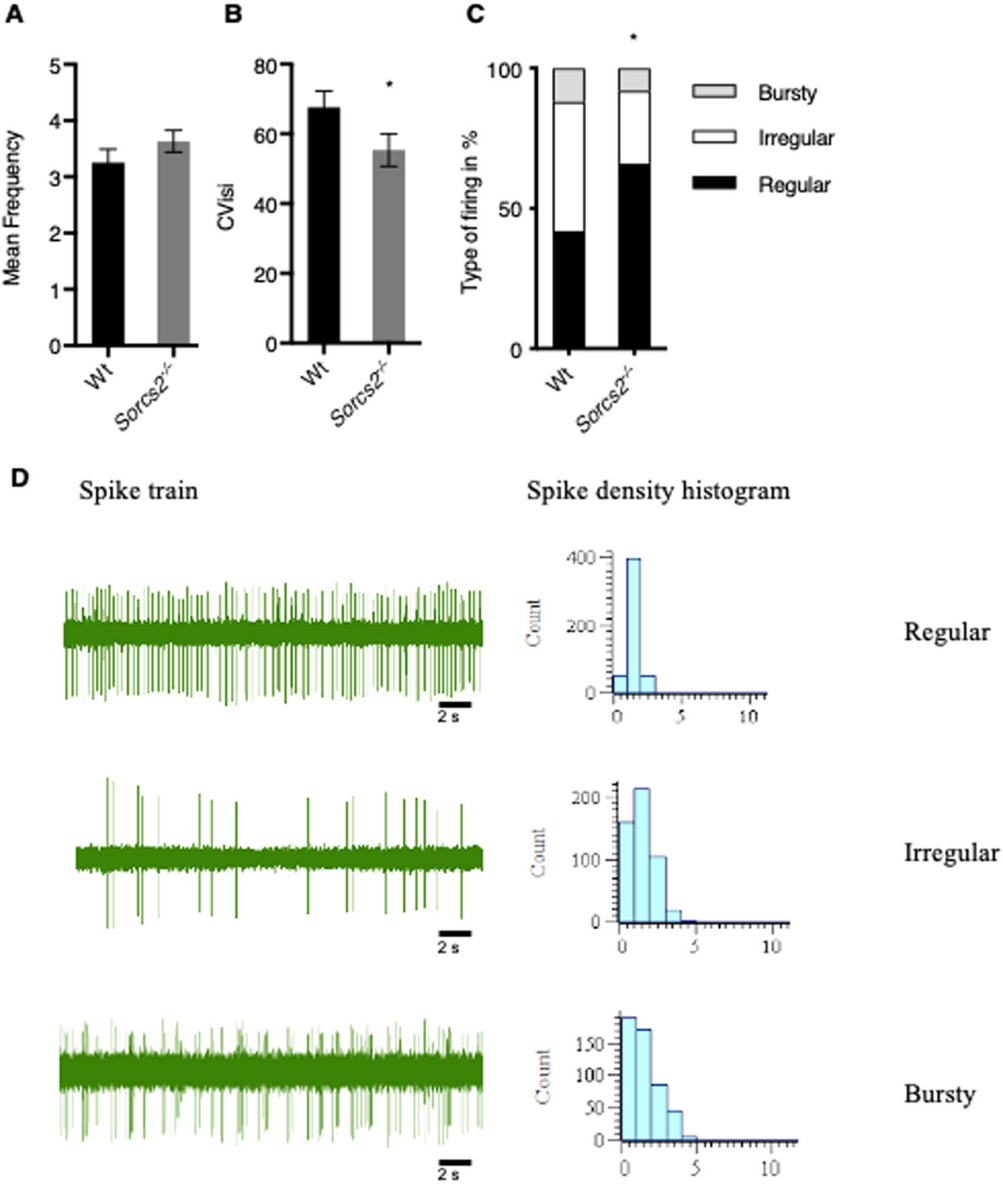
Dopaminergic neurons in the VTA in *Sorcs2*^*−/−*^ mice fire more regularly compared to Wt mice. **A** Dopaminergic neurons in the VTA fire with similar frequency in Wt and *Sorcs2*^*−/−*^ mice (*p* > 0.2, Students unpaired *t*-test). **B** lower CVisi in *Sorcs2*^*−/−*^ mice (*p* < 0.03, Students unpaired *t*-test). **C** *Sorcs2*^*−/−*^ mice have a higher population of regular firing neurons (Chi-square test, Χ^2^ = 6.59, df = 2, *p* < 0.003). **D** Representative spike density histogram (right column) of dopaminergic neurons in the VTA, constructed from spike trains (left column). Neurons were either classified into regular (upper row), irregular (middle row), or bursty (lower row). 48 neurons were recorded in Wt mice (from 10 animals) and 63 neurons recorded in *Sorcs2*^*−/−*^ mice (from 12 animals). * *p* < 0.05.

As previous studies in *Sorcs2*^*−/−*^ mice have shown dopaminergic hyperinnervation we speculated that there might be fundamental alterations in dopaminergic neurotransmission in *Sorcs2*^*−/−*^ mice. To test this hypothesis, we performed in vivo electrophysiological single-unit recordings of dopaminergic neurons in the VTA of wild type (Wt) and *Sorcs2*^*−/−*^ mice. Analysis of the mean firing rate of all recorded neurons showed no significant difference between Wt and *Sorcs2*^*−/−*^ mice (3.3 ± 0.2 vs. 3.6 ± 0.2 Hz, Student’s unpaired *t*-test t(110) = 1.3 *p* > 0.2, Fig. 1A). To quantify temporal coding of spike trains, we utilized the variation coefficient of the interspike interval (CVisi). The CVisi value was significantly lower in *Sorcs2*^*−/−*^ mice compared to Wt mice indicating more regular firing in the *Sorcs2*^*−/−*^ mice (55.4 ± 4.7 vs. 67.6 ± 4.7, Mann Whitney test, for Wt (*Mdn* = 70.93) and for *Sorcs2*^*−/−*^ (*Mdn* = 46.77), *U* = 1150, *p* < 0.03, Fig. 1B). Spike density histograms and autocorrelograms constructed from spike trains were further used to qualitatively classify the firing pattern of each neuron into regular, irregular or bursty firing (Fig. 1C, D). In agreement with the reduction in CVisi, a significant change in the firing pattern distribution was observed between Wt and *Sorcs2*^*−/−*^ mice (X^2^(2) = 12.07, *p* < 0.003). Thus, a lower proportion of neurons exhibited irregular firing in *Sorcs2*^*−/−*^ mice (26%) compared to Wt mice (46%), while a higher proportion of neurons exhibited regular firing (66%) in *Sorcs2*^*−/−*^ mice compared to Wt mice (42%). Together, these findings show altered activity of VTA dopaminergic neurons in *Sorcs2*^*−/−*^ mice compared to Wt mice.

### *Sorcs2*^*−/−*^ mice exhibit reduced sucrose intake

**Fig. 2:**
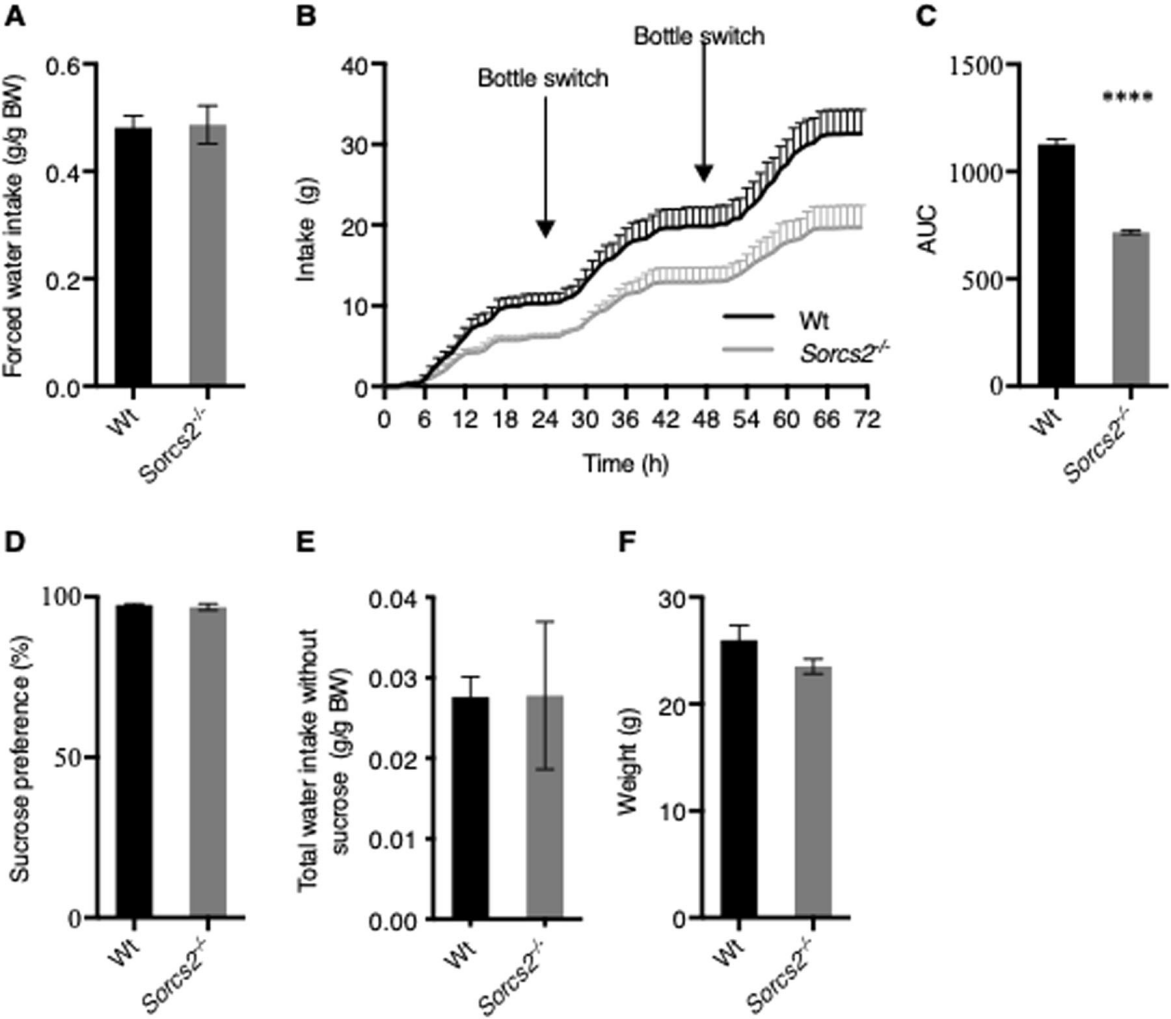
*Sorcs2*^*−/−*^ mice have lower sucrose intake. **A** Identical water intake in Wt and *Sorcs2*^*−/−*^ mice during a period of 48 h when water was the only drinking source (Student’s unpaired *t*-test, *p* > 0.9). **B**, **C** Cumulative intake of 4% sucrose solution of Wt and *Sorcs2*^*−/−*^ mice in a two-bottle test paradigm. Reduced consumption of sucrose-containing solution in *Sorcs2*^*−/−*^ mice compared to Wt mice displayed as AUC (*p* < 0.0001, Student’s unpaired *t*-test). **D** Sucrose preference (Student’s un-paired *t*-test *p* > 0.5). **E** Water intake during the three days of experiment (*p* > 0.15, Student’s unpaired *t*-test). **F** The weights of Wt and *Sorcs2*^*−/−*^ mice were not significantly different (*p* > 0.16, Student’s unpaired *t*-test). 4 mice of each genotype were tested. **** *p* < 0.0001.

It has previously been shown that altered dopaminergic neurotransmission can change sucrose preference in mice. Thus, a two-bottle sucrose test paradigm to examine anhedonia-like behavior and sucrose reward was conducted in Wt and *Sorcs2*^*−/−*^ mice. The mice first underwent a period of habituation for 48 h in the presence of two identical water bottles. During this period (forced water intake), water consumption was similar for the two genotypes (Student’s unpaired *t*-test, t(6) = 0.12, *p* > 0.9, Fig. 2A). However, when one bottle was exchanged for a sucrose-containing solution *Sorcs2*^*−/−*^ mice showed significantly lower intake of the sucrose-containing solution compared to Wt mice. Calculated as the area under the curve (AUC) the intake was 36% lower in *Sorcs2*^*−/−*^ mice compared to Wt mice (718.5 versus 1127, Student’s unpaired *t*-test, t(6) = 15, *p* < 0.0001, Fig. 2B, C), despite similar body weight (Student’s unpaired *t*-test, t(6) = 1.6, *p* > 0.16, Fig. 2F). This suggests impaired sucrose-associated reward in *Sorcs2*^*−/−*^ mice. Despite lower sucrose intake, the curve for the *Sorcs2*^*−/−*^ mice followed the same progression as the Wt mice, indicating that both genotypes learned the new position of the sucrose bottle equally well (Fig. 2B). Notably, the sucrose preference amounted to approximately 97% in both groups (Fig. 2D, Student’s unpaired *t*-test, t(6) = 0.59, *p* > 0.5). Intake of water devoid in sucrose was not different between genotypes too (Student’s unpaired *t*-test, t(6) = 0.017, *p* > 0.9, Fig. 2E), since in both groups it was reduced by ~ 95% compared to the volume consumed during the forced water intake. Hence, small differences in water intake (g/g BW) will cause relatively large variations. However, when compared to the overall solution intake these variations were negligible.

### Hyperactivity of *Sorcs2*^*−/−*^ mice is modulated by novelty

**Fig. 3:**
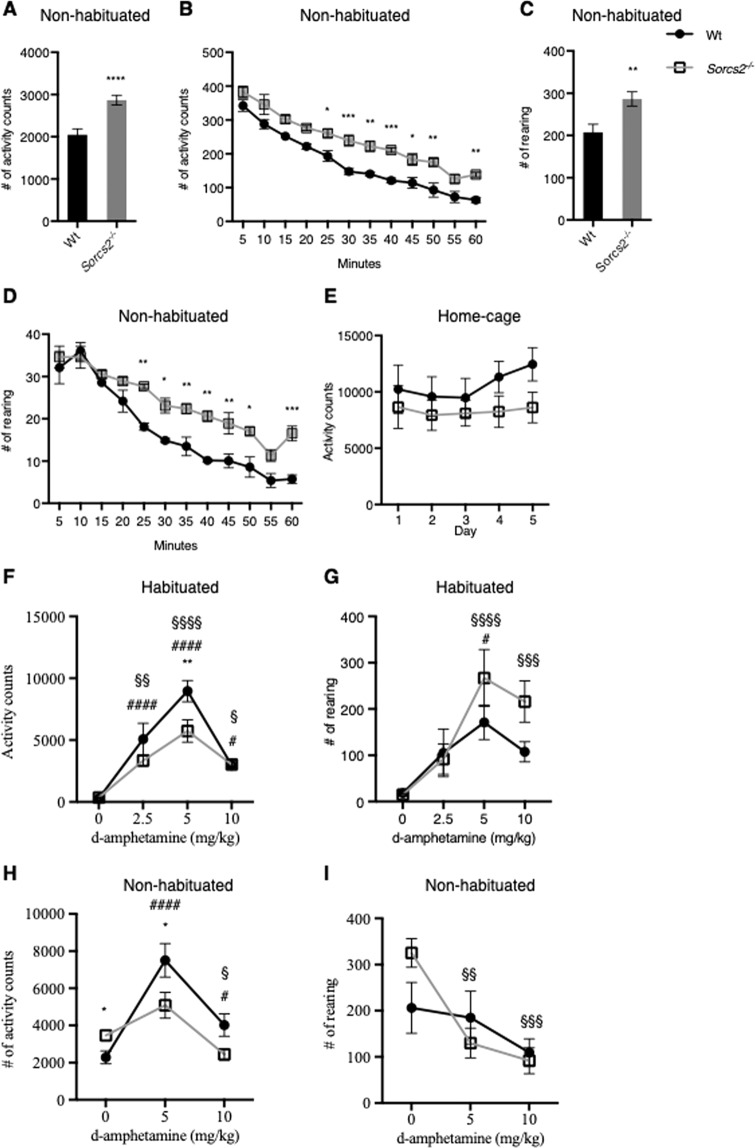
Behavioral effects of habituation, novelty, and amphetamine in *Sorcs2*^*−/−*^ mice. **A**, **B** *Sorcs2*^*−/−*^ mice are significantly more active (*p* < 0.0001, Student’s unpaired *t*-test) and **C**, **D** rear significantly more (*p* < 0.003, Student’s unpaired *t*-test) in a novel environment compared to Wt mice. **E** Wt and *Sorcs2*^*−/−*^ mice show normal activity in their home-cage (*p* > 0.4, two-way ANOVA). **F** Interaction between genotype and amphetamine dose on locomotor activity (*p* < 0.05, two-way ANOVA) **G** but not rearing (*p* > 0.2, two-way ANOVA) in habituated mice. **H** Interaction between genotype and amphetamine dose on locomotor activity (*p* < 0.007, two-way ANOVA) **I** but not rearing (*p* > 0.08, two-way ANOVA) in non-habituated mice was observed. In non-habituated mice, a significant increase in activity was observed in Wt mice when treated with 10 mg/kg compared to the vehicle group, while the activity decreased in *Sorcs2*^*−/−*^ mice (**H**, *p* < 0.03 for both genotypes, Student’s unpaired *t*-test). Data in **A**, **C** + **F**–**I** are presented as mean activity count/rearing for one hour per mouse ± S.E.M (*n* = 48 mice per group for **A** and **C** and 12 mice per group for **F**–**I**). Data for **B** and **D** are the corresponding data to **A** and **C**, respectively, in which activity/rearing is shown over time in 5 min intervals, Data in **E** are presented as mean activity count for 24 h per day per mouse ± S.E.M (*n* = 8 mice per group). Above the graph, it is stated if the mice were habituated or not to the test-cage prior to drug treatment. Asterisk indicates a significant difference between genotypes, while # and § indicate significant differences between the treated group and vehicle group within Wt and *Sorcs2*^*−/−*^ mice, respectively. *, # and § *p* < 0.05; **, ## and §§ *p* < 0.01; *** and §§§ *p* < 0.001; ****, #### and §§§§ *p* < 0.0001.

We monitored the basal locomotor activity in Wt and *Sorcs2*^*−/−*^ mice when subjected for one hour to a novel environment. *Sorcs2*^*−/−*^ mice showed a 40% increase in locomotor activity (Student’s unpaired *t*-test t(90.79) = 4.56, *p* < 0.0001, Fig. 3A) and a 38% increase in rearing (Student’s unpaired *t*-test t(91.84) = 3.11, *p* < 0.003, Fig. 3C) compared to Wt mice. Moreover, the *Sorcs2*^*−/−*^ mice continued to actively explore the test cage for a longer time period than Wt mice, suggesting reduced habituation to the novel environment, However, the decline in locomotor activity was similar between the two genotypes, as no interaction between genotype and activity was observed (mixed ANOVA F(11,66) = 0.83, *p* = 0.61), while both genotype and time had main effects (F(1,6) = 43.59, *p* = 0.0003, and F(11,66) = 83.83, *p* < 0.0001, respectively, Fig. 3B). In contrast, an interaction between genotype and time on rearing was observed (mixed ANOVA F(11,66) = 2.25, *p* < 0.05), and both genotype and time had main effects (F(1,6) = 126, *p* < 0.0001, and F(11,66) = 47.72, *p* < 0.0001, respectively) (Fig. 3D). This difference in activity appeared to be influenced by the exposure to a novel environment as no difference in locomotor activity was observed between Wt and *Sorcs2*^*−/−*^ mice when measured in their home-cage measured over 5 consecutive days (Mixed ANOVA F(4,48) = 0.97, *p* = 0.43, Fig. 3E).

### Amphetamine response of *Sorcs2*^*−/−*^ mice is affected by novelty

Direct or indirect stimulation of dopamine receptors affects locomotor activity in mice. Amphetamine acts on both dopamine release, and on the dopamine transporter, DAT, making the transporter working in reverse i.e. DAT pumps dopamine out to the synaptic cleft instead of taking it up^27,28^ altogether resulting in increased synaptic dopamine.

To study the role of test cage habituation in the locomotor response to amphetamine, we subsequently exposed mice to the test cage for one hour prior to a single acute dose of amphetamine (2.5, 5, or 10 mg/kg). Both amphetamine concentration and genotype had main effects on locomotor activity (Fig. 3F, Two-way ANOVA, F(3,87) = 37.95, *p* < 0.0001, and F(1,87) = 6.6, *p* < 0.02). A significant drug × genotype interaction on locomotor activity was observed (Two-way ANOVA, F (3,87) = 2.8, *p* < 0.05). A Sidak´s *post hoc* analysis showed a significantly reduced response in *Sorcs2*^*−/−*^ mice compared to Wt (Mean Diff = 3220, SE of diff = 936) following 5 mg/kg (*p* < 0.005), whereas a similar increase in activity was observed in both genotypes following a dose of 2.5 mg/kg amphetamine (Mean Diff = −4782, SE of diff = 936, *p* < 0.001 for Wt and Mean Diff = −2994, SE of diff = 936, *p* < 0.01 for *Sorcs2*^*−/−*^). Only amphetamine concentration had a significant main effect on rearing (Two-way ANOVA, F (3,87) = 10.9, *p* < 0.0001). In both genotypes 5 mg/kg of amphetamine induced significant increase in rearing (Tukey’s post hoc analysis, Mean Diff = −153, SE of diff = 52 *p* < 0.03 for Wt and Mean Diff = −252, SE of diff = 52, *p* < 0.0001 for *Sorcs2*^*−/−*^, Fig. 3G), while no interaction between amphetamine dose and genotype on rearing was observed (Two-way ANOVA, F(3,87) = 1.50, *p* > 0.2).

In a different experiment, mice were introduced to the test cage without prior habituation and immediately after administration of a single acute dose of vehicle or amphetamine (5 or 10 mg/kg). Here, both genotype and drug dose had main effects on locomotor activity (Two-way ANOVA, F(1,66) = 4.075, *p* < 0.05, and F(2,66) = 21.83, *p* < 0.0001, respectively, Fig. 3H). Moreover, an interaction between genotype and amphetamine dose on locomotor activity was observed (Two-way ANOVA, F(2,66) = 5.48, *p* < 0.007). *Sorcs2*^*−/−*^ mice still responded less to amphetamine compared to Wt when treated with 5 mg/kg (Sidak´s post hoc analysis, Mean Diff = −2420, SE of diff = 804.7, *p* < 0.02). However, in contrast to habituated animals (Fig. 3F), the activity of *Sorcs2*^*−/−*^ mice was reduced at 10 mg/kg amphetamine compared to the vehicle group, although significance was only revealed with a student unpaired *t*-test, t(22) = 2.54, *p* < 0.02), but not with a Sidak’s post hoc analysis (Mean Diff = 1017, SE of diff = 804.7, *p* = 0.2). However, the same dose significantly increased the activity in Wt mice (Sidak’s post hoc analysis, Mean Diff = -1740, SE of diff = 804.7 *p* < 0.04). These results are consistent with previous findings^15^. The response was reminiscent to that observed in humans diagnosed with ADHD^29^. In the rearing paradigm, only the amphetamine dose had a main significant (Two-way ANOVA, F(2,66) = 8.4, *p* < 0.0006, Fig. 3I). These observations suggest that the locomotor activity-reducing effect of high doses of amphetamine in *Sorcs2*^*−/−*^ mice is critically affected by novelty-induced stress.

### *Sorcs2*^*−/−*^ mice display reduced DRD1 sensitivity

**Fig. 4:**
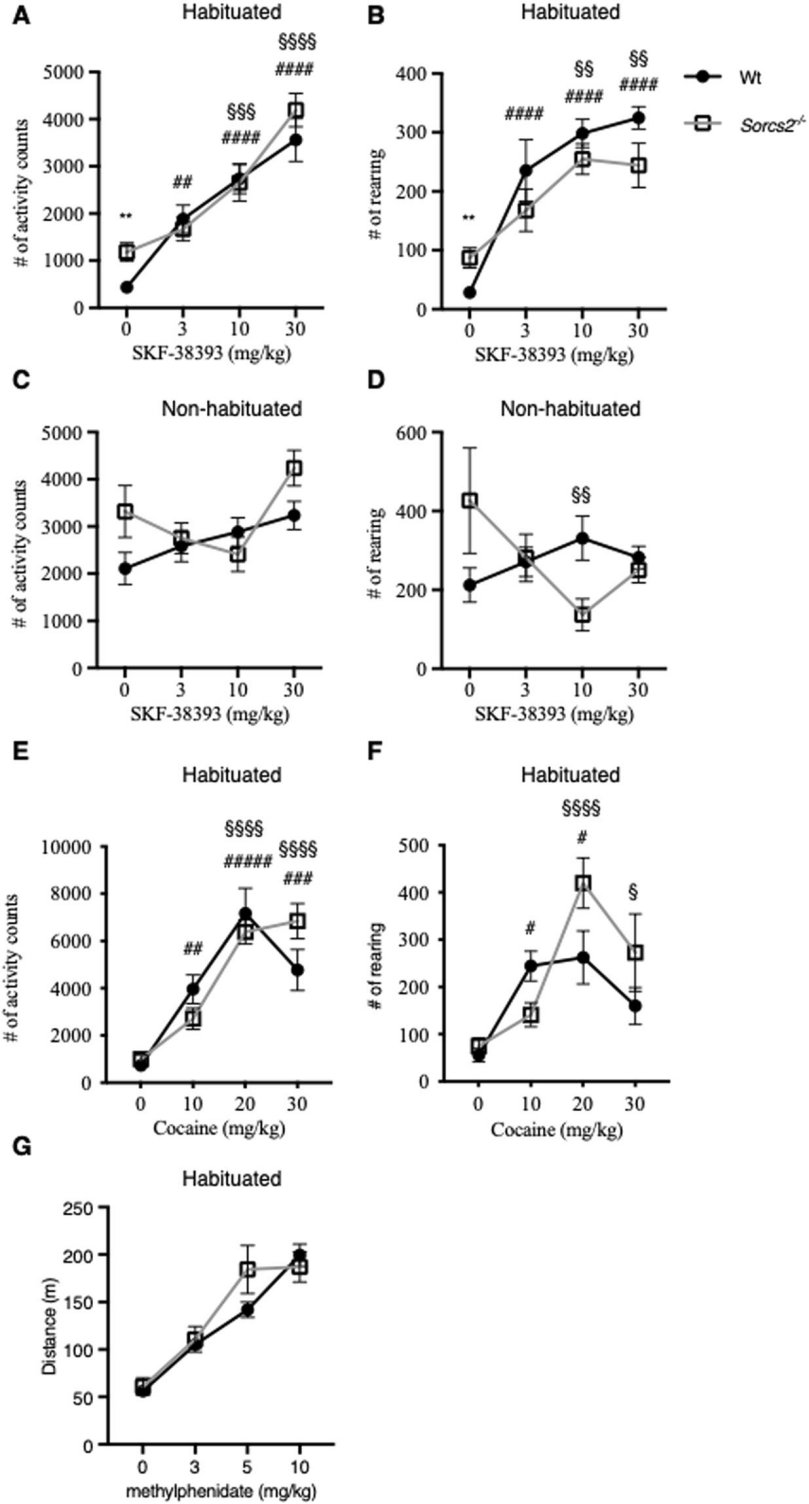
*Sorcs2*^*−/−*^ mice respond less to a DRD1 agonist (SKF-38393). **A**, **C** No interaction between genotype and dose of the DRD1 agonist SKF-38393 on locomotor activity (*p* > 0.3, two-way ANOVA) in neither habituated nor non-habituated mice (*p* > 0.3 and *p* > 0.09, respectively, two-way ANOVA), **B**, **D** while a significant interaction between dose of SKF-38393 and genotype on rearing was observed in both habituated and non-habituated mice (*p* < 0.03 for both setups, two-way ANOVA) was observed. Post hoc analysis showed that *Sorcs2*^*−/−*^ mice require higher doses of SKF-38393 to induce a response compared to Wt mice. **E** Wt and *Sorcs2*^*−/−*^ mice responded similar to cocaine-induced activity (*p* > 0.5, two-way ANOVA), **F** while, a significant interaction between cocaine dose and genotype on rearing was observed (*p* < 0.04, two-way ANOVA). **G** Wt and *Sorcs2*^*−/−*^ mice showed similar activity response to methylphenidate (*p* > 0.2, two-way ANOVA). Data are presented as mean activity count/rearing for one hour per mouse ± S.E.M, except for the methylphenidate data, where results are given as mean distance in meter ± S.E.M (*n* = 6–12 mice per group). Above the graph, it is stated if the mice were habituated or not to the test-cage prior to drug treatment. The * symbol indicates a significant difference between the two genotypes, while # and § indicate significant differences between the treated group and vehicle group in Wt and *Sorcs2*^*−/−*^ mice, respectively. *, § and # *p* < 0.05; **, ## and §§ *p* < 0.01; *** and §§§ *p* < 0.001; #### and §§§§ *p* < 0.0001.

Dopamine exerts its action by binding to one of five dopamine receptors (DR); DRD1-DRD5. While stimulation of DRD1 and DRD5 leads to activation of target neuron, activation of DRD2, DRD3, and DRD4 usually results in inhibition of the target neuron. The receptors have broad expression patterns in the brain and in the periphery. Especially the DRD1 and DRD2 are widely expressed in the brain. While all five receptors can be found postsynaptically on target neurons, DRD2 and DRD3 can also be found presynaptically on dopaminergic neuron. To study how the behavior of *Sorcs2*^*−/−*^ mice was influenced by postsynaptic dopamine receptor DRD1 activity, we tested the effect of the specific DRD1 agonist SKF-38393 (3–30 mg/kg) in habituated animals (Fig. 4A, B). SKF-38393 induced a dose-dependent increase in locomotor activity (Two-way ANOVA, F(3,40) = 35, *p* < 0.0001) and rearing (Two-way ANOVA, F(3,40) = 24, *p* < 0.0001) although no drug x genotype interactions on locomotor activity (Two-way ANOVA, F(3,40) = 1.2, *p* > 0.3) or rearing (Two-way ANOVA, F(3,40) = 2.2, *p* > 0.1) were observed. However, Tukey’s *post hoc* analysis showed that the activity of Wt mice increased with the lowest dose of SKF-38393, 3 mg/kg (Mean Diff = −1450, SE of diff = 442.5, *p* < 0.02), while a significant increase of activity in *Sorcs2*^*−/−*^ mice required 10 mg/kg (Mean Diff = −1470, SE of diff = 442.5, *p* < 0.01). This suggests that *Sorcs2*^*−/−*^ mice have reduced DRD1 receptor activity or sensitivity. We subsequently tested the response to SKF-38393 in non-habituated animals (Fig. 4C, D). SKF-38393 dose had a main effect on locomotor activity (Two-way ANOVA, F(3,40) = 4.1, *p* < 0.02) but not on rearing (Two-way ANOVA, F(3,40) = 0.6, *p* > 0.5). While no drug x genotype interaction was observed on locomotor activity (Two-way ANOVA, F(3,40) = 2.24, *p* > 0.09), a significant interaction between SKF-38393-concentration and genotype was observed on rearing (Two-way ANOVA, F(3,40) = 3.61, *p* < 0.03).

### Altered response to inhibition of the dopamine transporter in *Sorcs2*^*−/−*^ mice

To study how the mice responded to inhibition of synaptic dopamine reuptake, by blocking the dopamine transporter DAT, the response to cocaine in Wt and *Sorcs2*^*−/−*^ mice following habituation to the test cage was evaluated (Fig. 4E, F). The drug concentration had a main effect on both locomotor activity (Two-way ANOVA, F(3,81) = 31.10, *p* < 0.0001) and rearing (Two-way ANOVA, F (3,81) = 11.53, *p* < 0.0001). Genotype had no main effect on locomotor activity (Two-way ANOVA, F(1,81) = 0.02, *p* > 0.87) nor on rearing (Two-way ANOVA, F(1,81) = 1.96, *p* > 0.16). However, an interaction between drug dose and genotype on rearing was observed (Two-way ANOVA, F(3,81) = 2.95, *p* < 0.05) while an interaction between drug dose and genotype on locomotor activity was not significant (Two-way ANOVA,F(3,81) = 2.47, *p* = 0.068). A Sidak´s *post hoc* analysis revealed that while a dose of 10 mg/kg cocaine increased locomotor activity in Wt mice (Mean Diff = −3222, SE of diff = 977, *p* < 0.008), 20 mg/kg was necessary in *Sorcs2*^*−/−*^ mice (Mean Diff = −5385, SE of diff = 977, *p* < 0.0001). The same was observed for rearing where 10 mg/kg increased rearing significantly in Wt mice (Mean Diff = −188, SE of diff = 68, *p* < 0.05) while 20 mg/kg was required in *Sorcs2*^*−/−*^ mice (Mean Diff = −349, SE of diff = 66, *p* < 0.0001).

To test the activity of DAT further, the response to methylphenidate, a drug that acts on both DAT and the norepinephrine transporter NET, was evaluated in Wt and *Sorcs2*^*−/−*^ mice following habituation to the test cage (Fig. 4G). No interaction between methylphenidate dose and genotype on locomotor activity was observed in the mice (Two-way ANOVA, F(3,56) = 1.6, *p* > 0.2). Like for the treatment with cocaine, methylphenidate dose had a main effect on the locomotor activity (Two-way ANOVA, F(3,56) = 42, *p* < 0.0001).

### Increased DRD2 sensitivity in *Sorcs2*^*−/−*^ mice

**Fig. 5:**
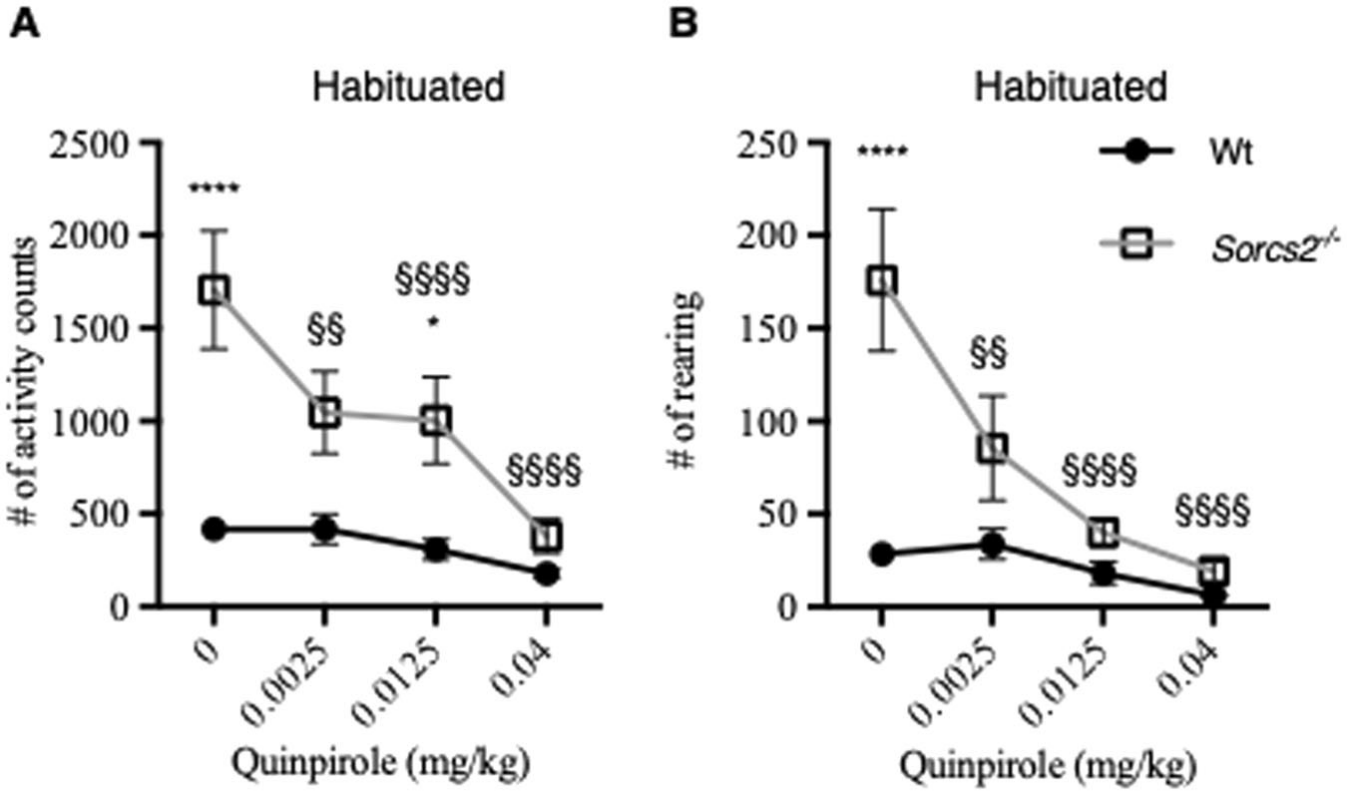
*Sorcs2*^*−/−*^ mice hyperactivity is normalized upon treatment with DRD2 agonist quinpirole. **A***. Sorcs2*^*−/−*^ mice respond differently to quinpirole-induced hypoactivity (*p* < 0.04, two-way ANOVA) and **B**. Quinpirole-induced rearing (*p* < 0.003, two-way ANOVA) compared to Wt mice. Data are presented as mean activity count/rearing for one hour per mouse ± S.E.M (*n* = 6 mice per group). Above the graph, it is stated if the mice were habituated or not to the test-cage prior to drug treatment. The * symbol indicates a significant difference between the two genotypes, while § indicates significant differences between the treated group and vehicle group in *Sorcs2*^*−/−*^ mice. * *p* < 0.05; §§ *p* < 0.01; **** and §§§§ *p* < 0.0001.

We next studied *Sorcs2*^*−/−*^ mouse behavior at the level of the presynaptic dopamine receptor DRD2, and tested the response to quinpirole after the mice had been habituated to the test cage (0.0125–0.04 mg/kg), a DRD2 agonist (Fig. 5A, B). Both genotype and the dose of quinpirole had main effects on activity (Two-way ANOVA, F (1,81) = 30.37, *p* < 0.0001 and F (3,81) = 6.245, *p* < 0.001, respectively). Significant interactions between genotype and quinpirole dose on hypolocomotor activity (Two-way ANOVA, F(3,81) = 3.0, *p* < 0.04) and rearing (Two-way ANOVA, F(3,78) = 5.3, *p* < 0.003) were observed. Quinpirole had a strikingly dose-dependent activity-reducing effect on *Sorcs2*^*−/−*^ mice, reaching the baseline Wt activity levels at 0.04 mg/kg quinpirole. Taken together, DA receptor modulators affecting the DRD1 and DRD2 had different response in Wt and *Sorcs2*^*−/−*^, while dopamine reuptake inhibition only showed minor differences between the genotypes. This suggests that *Sorcs2*^*−/−*^ mice have a perturbed balance of DRD1/DRD2 activity compared to Wt mice.

## Discussion

The dopaminergic system is essential for the control of locomotor activity, attention, the experience of reward as well as goal-directed behavior. Dopaminergic neurons fire in vivo in a spectrum of patterns ranging from regular pacemaker mode, to more irregular and bursty modes^30,31^. The regular and irregular firing modes represent the electrophysiological states correlating with tonic dopamine signaling, associated with extra-synaptic dopamine release. This tonic dopamine release, supplies a stable baseline level of extrasynaptic dopamine. In contrast, the bursty mode correlates with phasic dopamine signaling which is related to rapid, high amplitude, intra-synapatic dopamine release^32^. The phasic burst firing is believed to be involved in reward and goal-directed behavior^33,34^. To our knowledge, a shift in dopaminergic firing in the VTA from an irregular to a regular firing pattern as observed in *Sorcs2*^*−/−*^ mice, has not been reported before. Contrary, a shift from regular to a more irregular and bursty firing pattern in VTA has been shown in a rat and a mouse model mimicking symptoms associated with schizophrenia^16,24^. Interestingly, these rats and mice show increased sensitivity to amphetamine treatment^16,24^, i.e. opposite of what is observed in *Sorcs2*^*−/−*^ mice. Inhibition of dopaminergic firing in the VTA has previously been shown to reduce sucrose preference^35^. Although *Sorcs2*^*−/−*^ mice show the same preference for sucrose-containing water as Wt mice, the *Sorcs2*^*−/−*^ mice drink substantially less of the solution. One could speculate that while *Sorcs2*^*−/−*^ mice prefer to drink sucrose-containing solution when they are thirsty they do not get the same reward sensation as Wt mice. Therefore, *Sorcs2*^*−/−*^ mice stop drinking when they have quenched their thirst, while Wt might continue drinking. Similar behaviors have been reported for mice lacking DRD1^36^, DRD2^37,38^, and ionotrophic glutamate receptor signaling^39^. The finding is in accordance with our previous observation that *Sorcs2*^*−/−*^ mice also have reduced intake of alcohol in a two-bottle choice, a behavioral response that also is controlled by the dopaminergic system^40,41^.

Both DRD1 postsynaptic receptors and DRD2 autoreceptors are involved in long-term regulation of dopaminergic firing in VTA, but DRD2, in particular, is important for a robust regular firing pattern^42^. We here find that the locomotor response in *Sorcs2*^*−/−*^ mice to the DRD1 receptor agonist SKF-38393 was reduced. This suggests that *Sorcs2*^*−/−*^ mice display compromised DRD1 receptor activity^43^. Meanwhile, a significant interaction between treatment with the presynaptic DRD2 dopamine receptor agonist quinpirole and genotype was observed on locomotor activity and rearing. The inhibitory dopamine autoreceptors DRD2 are approximately six times more sensitive to dopamine agonists such as quinpirole than postsynaptic receptors^44,45^. Accordingly, a low dose of quinpirole induces hypoactivity due to stimulation of the presynaptic inhibitory DRD2. A role for DRD3 in the quinpirole-induced hypoactivity likely can be excluded since quinpirole-induced hypoactivity is similar in Wt and *DRD3*^*−/−*^ mice^46^. DRD2 overexpressing mice were also hyperactive compared to control mice^47^, suggesting that *Sorcs2*^*−/−*^ mice possibly display increased DRD2 autoreceptor activity compared to the Wt mice. The imbalance between DRD1 and DRD2 activity could be a consequence of the altered dopaminergic connectivity in the frontal cortex of the *Sorcs2*^*−/−*^ mice^15^. Interestingly, DRD1 expression can be regulated by brain-derived neurotrophic factor (BDNF) in the catecholaminergic cell line CAD^48^. We previously reported that SorCS2 binds tropomyosin receptor kinase B (TrkB), to facilitate signaling by its ligand BDNF^11^, and it is tempting to speculate that, perturbed BDNF signaling due to lack of SorCS2 may also influence the balance between DRD1 and DRD2 activity.

Dopaminergic transmission is highly influenced by novelty^49,50^. While the activity and rearing response in vehicle-treated Wt and *Sorcs2*^*−/−*^ mice showed some variance between expeiments, the response to novelty after drug treatment was very robust. In all non-habituated studies the locomotor activity was higher in *Sorcs2*^*−/−*^ mice, although this difference occasionally did not reach significance due to variation with the experiment. These variances can be due to unpredictable factors such as sudden noise in the behavioral room and change in caretakers as a consequence of vacation etc. To minimize the variance, the same experimenter made all activity studies and Wt and *Sorcs2*^*−/−*^ mice were always tested in parallel.

It is interesting that *Sorcs2*^*−/−*^ mice displayed hyperactivity when subjected to a novel environment but not in their home cage, and that the behavioral response of *Sorcs2*^*−/−*^ mice to amphetamine was highly influenced by novelty. Novelty-induced hyperactivity is also observed in other transgenic animals with altered dopaminergic system such as *DAT*^*−/−*^ and *DAT*^*+/−*^ mice^51,52^, *DRD1*^*−/−*^ mice^53,54^ and DRD2 overexpressing mice^47^. Moreover, *Sorcs2*^*−/−*^ mice seemed to habituate slower to the new environment than Wt mice. High novelty-seeking and reduced habituation to novel environments have been correlated with high basal levels of dopamine in striatum^23,55^ and are observed in DAT knockdown and *DRD1*^*−/−*^ mice^23,56^, suggesting that perturbation of dopaminergic functionality in general leads to an altered response to novelty. Intriguingly, individuals suffering from ADHD exhibit enhanced neural activity in response to novel but behaviorally irrelevant stimuli, as well as reduced habituation to familiar items, compared to unaffected individuals^57^.

The habituated *Sorcs2*^*−/−*^ mice displayed a blunted response to amphetamine compared to Wt mice. Such a response has also been observed in netrin-1-receptor-deficient mice^58^ and EphA5 overexpressing mice^59^ that are both characterized by altered dopaminergic connectivity. Interestingly, the paradoxical activity-reducing response to amphetamine observed in non-habituated *Sorcs2*^*−/−*^ mice is similar to the response observed in *DAT*^*−/−*^ mice. These mice display a lack of response to amphetamine and cocaine when habituated for two hours prior to drug treatment^52^, while an activity-reducing effect is observed if given after 30 min of habituation^60^. This attenuating effect of amphetamine in *DAT*^*−/−*^ mice is fully reversed by subsequent administration of a low dose of (+)-MK-801, a selective NMDA receptor antagonist, suggesting that the hypolocomotor effect of psychostimulants in *DAT*^*−/−*^ mice requires intact glutamatergic neurotransmission^61^. Remarkably, SorCS2 has been shown to control NMDAR trafficking and its insertion into the synapse in striatum and hippocampus and NMDAR-dependent glutamatergic synaptic plasticity is eliminated in *Sorcs2*^*−/−*^ mice^11,62,63^. This suggests that both the dopaminergic and glutamatergic systems may be involved in the ADHD-like behavior. *Sorcs2*^*−/−*^ mice showed no significant difference in response to methylphenidate, and only rearing was different in response to cocaine. As these drugs act by inhibiting the dopamine transporter, DAT, and norepinephrine transporter, NET, it suggests that there is no marked difference in the released amount of dopamine (or noradrenaline) compared to Wt mice.

A recent exome sequencing study in ADHD showed the presence of more than twice as many rare and disruptive variants among cases in a defined set of genes previously associated with ADHD from GWAS meta-analysis^64,65^. Interestingly, pathway analyses of the top candidates emerging from the published genome-wide associations studies have revealed an enrichment in risk genes involved in neurite outgrowth and axon guidance^66,67^, rendering it tempting to speculate that abnormal guidance of dopaminergic projections during development is a common underlying cause of ADHD, and that lack of SorCS2 could play a role in this abnormality. Interestingly, *SORCS3*, encoding a SorCS2 paralogue^68^, was among the first 12 loci discovered to be genome-wide significantly associated with risk of ADHD^69^. The reported odds ratio was 0.911 which indicates that the SORCS3 locus is protective. In analogy, a copy number variation (CNV) in an ADHD case resulting in the duplication of *SORCS3* and the related gene *SORCS1* has also been reported^70^. In all, the findings indicate a critical role for SorCS receptors in the development of ADHD.

In conclusion, our findings show that the lack of SorCS2 causes altered response to psychostimulants and altered dopaminergic firing patterns in the VTA in vivo. Moreover, lack of SorCS2 causes a reduced DRD1 sensitivity or activity and increased DRD2 autoreceptor activity or sensitivity. Remarkably, the response to agonists of these receptor systems is susceptible to novelty-induced stress. However, there are some limitations to the present study. We investigated the impact of SorCS2 deficiency on dopaminergic functionality using offspring from *Sorcs2*^*−/−*^ mice and using locomotor activity as readout for drug response. In the future, it would be interesting to use conditional knockout or siRNA to knock down Sorcs2 postnatally. Such studies could assess if the observed phenotype is developmental or not. Moreover, *Sorcs2* could be knocked down in specific regions to identify the brain regions involved in the observed phenotype. It would also be interesting to study the changes in dopaminergic receptors in a non-pharmacological manner, for example genetically manipulate the dopamine receptors in WT and *Sorcs2*^*−/−*^ mice or a more biochemical approach to study the levels of dopamine receptors such as ELISA. In brief, our findings provide important insight into the etiology of ADHD, and show for the first time how a lack of a risk gene that affects axon guidance in the developing dopaminergic system results in altered transmission and response to central stimulants in adults.
